# Antioxidative Defense and Gut Microbial Changes under Pollution Stress in *Carassius gibelio* from Bucharest Lakes

**DOI:** 10.3390/ijerph19127510

**Published:** 2022-06-19

**Authors:** Cristina F. Alistar, Ionela C. Nica, Mihai Nita-Lazar, Gabriela Geanina Vasile, Stefania Gheorghe, Alexa-Maria Croitoru, Georgiana Dolete, Dan Eduard Mihaiescu, Anton Ficai, Nicolai Craciun, Gratiela Gradisteanu Pircalabioru, Mariana Carmen Chifiriuc, Miruna S. Stan, Anca Dinischiotu

**Affiliations:** 1 Department of Biochemistry and Molecular Biology, Faculty of Biology, University of Bucharest, 91-95 Spl. Independentei, 050095 Bucharest, Romania; cristina.pelcaru@drd.unibuc.ro (C.F.A.); cristina.nica@drd.unibuc.ro (I.C.N.); nicolai.craciun@bio.unibuc.ro (N.C.); adin@bio.unibuc.ro (A.D.); 2 Research Institute of the University of Bucharest (ICUB), University of Bucharest, 050095 Bucharest, Romania; gratiela.gradisteanu@icub.unibuc.ro (G.G.P.); carmen.chifiriuc@bio.unibuc.ro (M.C.C.); 3 National Research and Development Institute for Industrial Ecology (ECOIND), 57-73 Drumul Podu Dambovitei, 060652 Bucharest, Romania; mihai.nita@incdecoind.ro (M.N.-L.); gabriela.vasile@incdecoind.ro (G.G.V.); stefania.gheorghe@incdecoind.ro (S.G.); 4 Department of Science and Engineering of Oxide Materials and Nanomaterials, Faculty of Chemical Engineering and Biotechnologies, University Politehnica of Bucharest, Gh. Polizu St. 1-7, 060042 Bucharest, Romania; alexa_maria.croitoru@upb.ro (A.-M.C.); dolete.georgiana@gmail.com (G.D.); dan.mihaiescu@upb.ro (D.E.M.); anton.ficai@upb.ro (A.F.); 5 National Centre for Food Safety, University POLITEHNICA of Bucharest, Splaiul Independentei 313, 060042 Bucharest, Romania; 6 National Centre for Micro- and Nanomaterials, University POLITEHNICA of Bucharest, Splaiul Independentei 313, 060042 Bucharest, Romania; 7 Academy of Romanian Scientists, 3 Ilfov Street, 050045 Bucharest, Romania; 8 Department of Microbiology, Faculty of Biology, University of Bucharest, 050095 Bucharest, Romania

**Keywords:** aquatic pollution, oxidative stress, fish, Bucharest, microbiome

## Abstract

Fish are able to accumulate by ingestion various contaminants of aquatic environment, with negative consequences on their intestine, being continuously threatened worldwide by heavy metals, pesticides and antibiotics resulted from the human activities. Consequently, the health of other species can be affected by eating the contaminated fish meat. In this context, our study aimed to perform a comparison between the changes in intestine samples of *Carassius gibelio* individuals collected from different artificial lakes in Bucharest (Romania), used by people for leisure and fishing. The presence of various metals, pesticides and antibiotics in the gut of fish was assessed in order to correlate their accumulation with changes of antioxidative enzymes activities and microbiome. Our results showed that fish from Bucharest lakes designed for leisure (Chitila, Floreasca and Tei lakes) have an increased level of oxidative stress in intestine tissue, revealed by affected antioxidant enzymes activities and GSH levels, as well as the high degree of lipid peroxidation, compared to the fish from protected environment (Vacaresti Lake). Some heavy metals (Fe, Ni and Pb) and pesticides (aldrin and dieldrin) were in high amount in the gut of fish with modified antioxidative status. In conclusion, our study could improve the knowledge regarding the current state of urban aquatic pollution in order to impose several environmental health measures.

## 1. Introduction

Water pollution occurs when harmful substances, often chemicals, as well as microorganisms, contaminate rivers, lakes and other water bodies, degrading water quality that becomes toxic to the environment. Water represents the solvent able to dissolve most of all chemical substances on Earth and as a consequence can be easily polluted.

In urban areas, the rain and melting snow rapidly run off into lakes and rivers through drain ways and storm sewers. This urban runoff contains insecticides, herbicides, heavy metals, emerging pollutants from trash and pet wastes.

All over the world, the urban anthropogenic activities generate wastes rich in heavy metals, that are discharged in the water bodies, accumulated in sediments and biomagnified through the food chain, with ecological consequences for invertebrates, fish and humans [1]. Apart these inputs, the natural ones include geological and pedological processes, due to natural weathering [2].

Among the water contaminants, pesticide play an important role in the control of pests, not only in agriculture, but also in urban areas. Herbicides, insecticides, fungicides, and rodenticides are used extensively and intensively in urban environment, that are runoff in water bodies [3]. Moreover, antibiotics are widely used to stop infections caused by bacteria, in animal husbandry and aquaculture. Their consumption increased tremendously in the last twenty years and a percent of about 30–90% is discharged in the environment in their native form or as metabolites [4]. Once entered the environment, antibiotics alter microbial communities and generate drug resistance in environmental bacteria via antibiotic resistance genes [5]. In this context, Swiacka et al. [6] realized an extensive review related to the presence of different pharmaceuticals and their metabolites (antibiotics, analgesics, antidiabetics, antidepressants, NSAIDS, etc.) and concluded that these agents can be found in the aquatic media (sewage sludge, water or in the aquatic organisms) in a wide range of concentration and different ratios, from ppb to ppm level, their biodegradation rates being very different.

In urban areas, water surfaces are most often polluted, with many negative consequences, affecting the living organisms (including fish), by generating a toxic environment, which can lead to alteration of their vital functions, and even death. Numerous studies on fish have suggested that high levels of pollution cause oxidative stress in the tissues of various organs, including the gut through the contact with ingested, contaminated water [7,8,9]. Recently, it was demonstrated that anthropogenic waste in the aquatic environment has an impact on the gut microbiome composition in fish, which affects the health state of fishes from physiological and immunological point of view [10].

Taking into consideration that leisure lakes in urban places can be threaten by pollution, our study was aimed to evaluate the presence of several metals, organochlorine and organophosphorus pesticides and antibiotics in the gut tissue of *Carassus gibelio* fish from leisure lakes in Bucharest, and the generation of oxidative stress, DNA lesions and antibiotic resistance genes in these samples. Within this evaluation, three leisure lakes used by people for fishing (Chitila, Floreasca and Tei) from Bucharest, Romania, were selected to collect fish and to compare with the same specimen from Vacaresti Lake, a protected area in the same city.

## 2. Materials and Methods

### 2.1. Ethical Statement

Animal care protocols and experimental procedures were performed in accordance with the Guide for Use and Care of Laboratory Animals (European Communities Council Directive 1986), Organization for Economic Co-operation and Development (OECD) recommendations regarding reduction of animal suffering and number of animals sacrificed, and the existing laws in Romania: Law no. 206/2004 on good conduct in scientific research, technological development and innovation, Law no. 471/2002 regarding the approval of the Romanian Government Ordinance no. 37/2002 for the protection of animals used for scientific or other experimental purposes, Law no. 462/2001 on the regime of protected natural areas, conservation of natural habitats, wild flora and fauna.

### 2.2. Test Organisms and Experimental Procedure

**Figure 1:**
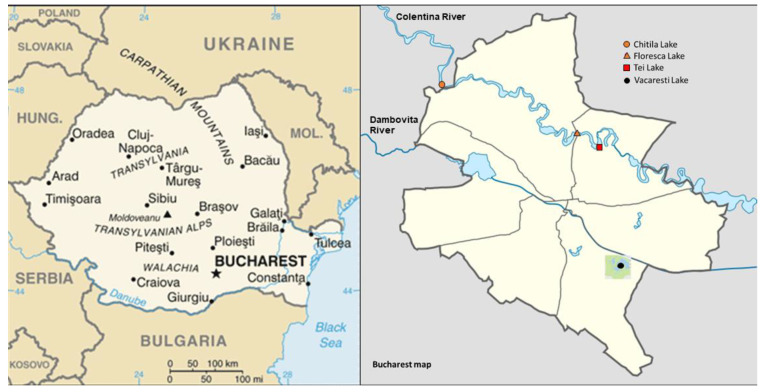
Maps of Romania (**left**) and Bucharest (**right**) showing the four lakes (Chitila, Floreasca, Tei and Vacaresti) selected to collect the fish for further experiments.

Originally from Siberia, Prussian carps (*Carassius gibelio)* are highly invasive fish species in different lakes, ponds, and slow-moving rivers throughout Europe, North America, and Asia. *Carassius gibelio* is also one of the most common fish in the Romanian lakes and is inevitably exposed to all kind of pollutants in the water as the individuals are omnivorous and feed on plankton, invertebrates, plant material and detritus [11]. The tests performed in this experimental study were conducted on individuals from 4 different populations: Chitila, Floreasca, Tei and Vacaresti lakes (from Bucharest, Romania) (Figure 1). These anthropic lakes were selected due to their closeness to hospitals (Floreasca Emergency Hospital, Matei Bals Hospital) for Floreasca and Tei lakes, and to pharmaceuticals distribuitors (A & D Pharma Mediplus) for Chitila Lake. On the other hand, Vacaresti lake was selected as it is a protected environment by Romanian authorities (over 100 species of wild birds and animals live in this area). Chitila, Floreasca and Tei lakes are located in the north of Bucharest, the capital of Romania, on the Colentina river, but due to its low flow (between 2.44 m/s și 0.12 m/s) the whole chain of lakes in this area is fed by the Ialomita river through the Bilciuresti-Ghimpati Chanel. Because the lakes must also allow the safe evacuation of high flows transiting from upstream (Chitila) to downstream (Floreasca, Tei), Chitila and Floreasca lake communicate through Herastrau lock, while Floreasca and Tei lakes are separated by a dam. Vacaresti lake is located in the south of Bucharest and was formed from an old bifurcation of the Dambovita River, the main source of drinking water for the citizens of Bucharest. It was filled only once with water from Dambovita river, but the costs for pumping the water were very high. Obviously, Vacaresti lake does not communicate with the other three lakes in the Colentina river valley. Seven specimens were collected from each sampling site (28 individuals, 7 fish/area). The fish were bought in August 2019 from authorized fishermen, and within the Delta Vacaresti Natural Area (Vacaresti Lake), an authorization was obtained for the collection of specimens for the purpose of scientific research, and not for water analysis which is under national regulation. The levels of contamination are already known [12]. According to the Lakes, Parks and Leisure Administration in Bucharest (ALPAB), especially in the case of Floreasca and Tei lakes, the pH is within normal limits, exceeding the value of 8.5 by only 0.02 and 0.38, respectively, and the ammonium, nitrites and nitrates levels are also within the normal limits. However, in the case of the other lakes from Bucharest, there are exceedances of the maximum allowed value for heavy metals, such as cadmium, zinc, copper and lead [12].

Each individual was weighed and measured after a first examination. The mean values ± standard errors of the size and weight of the individuals selected for the study can be found in Table 1. Gut tissue samples were collected and kept in the freezer at −80 °C for chemical, biochemical and molecular biology tests.

### 2.3. Determination of Metal Concentrations

Multielement Certified Reference Materials (CRM), standard solution of 28 elements, 100 mg/L (CPAChem, Bogomilovo, Bulgaria and unielement CRMs for Ca, Mg, Na and K 10 g/L each (Certipur, Merck KGaA, Darmstadt, Germany), ultra-trace nitric acid (69%, Supelco, Darmstadt, Germany) and ultrapure water for trace analysis (Milli-Q, IQ 7005, Millipore, Merck, Germany) were used for the calibration curves. For pre-treatment of fish tissue were used ultra-trace nitric acid and supra-pure hydrogen peroxide (30%, Supelco, Germany).

Freeze Dryer Hyper Cool HC3110 (LabTech, Sorisole, Italy) and Microwave Digestion System Ethos Up (Milestone, Sorisole, Italy) were used for pretreatment of the fish tissues. The metals concentration was quantified with an ICP-EOS AVIO 500 Spectrometer (Perkin Elmer, Waltham, MA, USA).

For multielement simultaneous determination (Cd, Cr, Cu, Fe, Mn, Ni, Pb and Zn) the calibration curve was set between 0.1 mg/L and 0.5 mg/L (axial view of the plasma), while for Ca, Mg, Na and K, the curve was plotted in the range of 5–50 mg/L (radial view of the plasma).

Fish tissues were freeze-dried at −110 °C for 24 h to 36 h. Pooled samples from 7 individuals per lake were obtained by weighing 0.10 g to 0.30 g mass, and a mixture of reagents was added (9 mL ultra-trace nitric acid and 1 mL hydrogen peroxide). The applied microwave program included three steps: step 1—raising the temperature to 200 °C for 15 min with a maximum power of 1800 W; step 2—maintaining the temperature for 15 min at 200 °C, and finally, step 3—15 min for cooling. The solutions were filtered and brought to 25 mL with ultrapure water.

### 2.4. Determination of Pesticide Residues

Both organochlorine (OC) and organophosphorus (OP) pesticides residues were evaluated in fish samples (gut) from different lakes. OC and OP were mixed together for the analysis of the fish samples by gas chromatography-mass spectrometry (GC-MS). The extraction and clean-up were done based on the sample preparation method for pesticides. Pooled samples from 7 individuals per lake were obtained by blending in an electric blender/grinder to obtain a homogeneous sample. The weight of each sample ranged between 1 to 5 g. Each sample was mixed and dried with anhydrous sodium sulphate and extracted three times with 25 mL of hexane (the remained solid mass was kept for the antibiotic extraction). The reunited pesticide extract was then filtered through a pre-rinsed chromatographic column containing 1:3 Florisil and anhydrous sodium sulphate (the clean-up step). The extract was evaporated with a Rotary evaporator (Hei-vap Advantage) to 1 mL, and the pesticide content was determined by GC-MS analysis. The fish samples were analyzed in triplicate.

Further, six-point calibration curve was constructed using pesticide standard solution (100 µg/L) with concentrations ranging from 5 µg/L to 100 μg/L, equivalent to 0.001 mg/kg and 0.02 mg/kg. The limit of detection (LOD) value for the pesticides residues was 1 µg/L, equivalent to 0.2 ng/kg of dry sample.

#### GC-MS Analysis

An Agilent Technologies 7890B GC System equiped with a mass spectrometer Q-TOF 7200 and an analytical column HP-5MS Ultra Inert (30 m long × 0.25 µm internal diameter × 0.25 μm film thickness) was used for analysis. The operating condition of OC and OP pesticides were pulsed splitless injection mode, 270 °C injector and 250 °C interface temperature, 0.3 min sampling time, helium gas as a carrier with flow rate 2 mL/min, 17 mL/min total flow rate, 1 mL/min column flow, 1 mL/min purge flow and injection volume of 1.8 μL. The temperature was programmed from an initial value of 70 °C, ramped to 150 °C at 15 °C/min, and to 235 °C at 2 °C/min, and was increased to 390 °C at 30 °C/min for 15 min and total run time was 68 min. The mass spectrometer Q-TOF 7200 was operated in the electron ionization (EI) mode, 70 eV with a mass range between 41–850 amu. Chromatograms and spectra were recorded and processed using MassHunter WorkStation software and the spectra library used was Mass Spectral Search Program v.2.0d, NIST 2005, Washington, DC, USA.

### 2.5. Determination of Antibiotics Residues

For the extraction and determination of antibiotics from the fish samples, the remaining solid mass after the pesticide extraction was dried in the oven at 35 °C for 24 h and transferred in a 50 mL centrifuge tube. Each sample was extracted two times in a row with 20 mL of methanol. Every time the mixture was vortexed for 10 min, followed by centrifugation for 10 min at 6000 rpm. The supernatant was transferred in a beaker for clean-up step. The reunited antibiotic extract was then filtered through a pre-rinsed chromatographic column containing Florisil. The extract was evaporated with a Rotary evaporator (Hei-vap Advantage) to 1 mL, and the antibiotic content was determined by liquid chromatography-mass spectrometry (LC-MS) analysis. The fish samples were analyzed in triplicate.

Linear calibration curves at seven levels of fortification with concentrations ranging from 10 µg/L to 150 μg/L were prepared in ultrapure water by adding known quantities of standard mixture solution (1000 μg/L). The limit of quantification (LOQ) values for the antibiotics were equivalent to 0.004 μg/g and 0.06 μg/g of dry sample. The limit of detection (LOD) value for the antibiotics was 5 µg/L, equivalent to 0.002 ug/g of dry sample.

#### LC-MS Analysis

Analyses were performed on Agilent Technologies 6540 UHD Accurate-Mass Q-TOF LC/MS equipped with a reversed-phase Zorbax Eclipse C18 (Agilent, 50 × 4.6 mm, 2.7 μm particle size). Mobile phases were 95% water (A) and 5% acetonitrile (B). Antibiotics were separated following gradient program: initial conditions were 95% A, then gradient was from 100% B to 5% B in 5 min and finally, solvents were maintained to 95% A and 5% B for 4 min. The total run time was of 14 min. Column temperature was 60 °C; flow rate was 0.15 mL/min. The sample injection volume was set at 5 μL. The mass spectrometer used was a Q-TOF system equipped with a Dual ESI ion source operated in positive ionization mode. The operating parameters were ion spray voltage 5300 V, drying gas, 7 L/min, nebulizer gas 21 psig and probe temperature 300 °C. For each compound, acquisition rate 1.1 spectra/s, 909.1 ms/spectrum.

### 2.6. Biochemical Analyses

#### 2.6.1. Preparation of the Total Protein Extracts

Total protein extracts (EPT) were obtained according to the protocol developed in our laboratory [13]. One hundred mg of gut tissue of each fish was added to 1 mL of 0.1 M TRIS-HCl buffer, 5 mM EDTA, pH = 7.4, resulting in a ratio of 1:10 (m/V); the disruption of the cell membranes was performed with a RETSCH MM301 ball homogenizer during two homogenizations of 2 min each, using a frequency of 30 vibrations per second, with 5 min rest on ice between the two homogenization cycles. The resulting homogenate was centrifuged at 8000× *g*, 30 min, 4 °C to remove cell debris. The supernatant was collected and used for subsequent biochemical tests. The protein concentration in the extracts was determined by Lowry method using bovine serum albumin as standard and the results were expressed in mg/mL.

#### 2.6.2. Assessment of Antioxidant Enzyme Activities

Superoxide dismutase (SOD) activity was determined according to the spectrophotometric method described by Paoletti et al., which is based on the ability of SOD to catalyze the reaction of transformation of the superoxide anion into molecular oxygen and hydrogen peroxide, in the presence of EDTA, MnCl_2_ and β-mercaptoethanol [14]. The reaction rate is determined by the decrease in absorbance at 340 nm due to NADH oxidation. Measurement of catalase (CAT) activity was performed according to the spectrophotometric method described by Aebi [15] by monitoring the decrease in absorbance of H_2_O_2_ at 240 nm. Glutathione S-transferase (GST) activity was tested according to the method of Habig et al. [16] by quantifying the conjugation rate of 1-chloro-2,4-dinitrobenzene (CDNB) with GSH. This was achieved by increasing the absorbance at 340 nm. Glutathione peroxidase (GPx) activity was tested according to the method described by Beutler [17] by monitoring the absorbance changes at 340 nm determined by a coupled reaction with glutathione reductase that catalyzed the conversion of NADPH to NADP^+^. Glutathione reductase (GR) activity was evaluated using the Golberg and Spooner’s method [18], which involves a mix of 0.1 M phosphate buffer (pH 7.8) with 33 mM of oxidized glutathione (GSSG) and 2 mM NADPH. The final results were calculated as specific enzymatic activities (units/mg of protein).

#### 2.6.3. GSH Level Measurement

Reduced glutathione (GSH) level was assessed using Sigma’s Glutathione Assay Kit (Sigma-Aldrich, St. Louis, MO, USA) after the proteins from the samples were removed by precipitation with a 5% sulfosalicylic acid solution (Sigma-Aldrich) (1:1) followed by 10 min centrifugation at 10,000 rpm and 4 °C. The principle of this colorimetric method consists in the oxidation of GSH by the sulfhydryl reagent 5,5′-dithio-bis (2-nitrobenzoic acid) (DTNB) which leads to the formation of the yellow derivative 5′-thio-2-nitrobenzoic acid (TNB) with a maximum absorption at 412 nm. Finally, GSH levels were calculated as nmols/mg protein by reference to the protein content determined using the Lowry method.

#### 2.6.4. Lipid Peroxidation

The malondialdehyde (MDA) level was assessed as a marker of lipid peroxidation by mixing 0.2 mL of total protein extract properly diluted with 0.7 mL of 0.1 N HCl solution, followed by an incubation at room temperature for 20 min. Subsequently, a volume of 0.9 mL of 0.025 M thiobarbituric acid (TBA) was added and the mixture was incubated for 65 min at 37 °C in order to allow the TBA-MDA products formation. a standard solution of 1 mM 1,1,3,3-tetramethoxypropane (malondialdehyde) was used, of which dilutions in 0.1 N HCl were made. Further, the MDA-TBA adducts were quantified fluorometrically (excitation/emission wavelength = 520/549 nm) and the relative fluorescence units recorded using the Jasco FP-6300 fluorimeter (FP-750 Spectrofluorometer, Jasco, Tokyo, Japan) were converted to nmols of MDA using a 1,1,3,3-tetramethoxypropane standard curve. The degree of lipid peroxidation was determined by reporting the MDA level from each sample to the corresponding protein concentration (nmols MDA/mg protein).

### 2.7. Molecular Biology Assays

#### 2.7.1. Quantification of 5-Methylcytosine

Measurements of 5-methylcytosine (5-mC) levels from the gut samples were carried out by an enzyme-linked immunosorbent assay (ELISA). For this measurement, the gut samples were pooled for all seven individuals of the same lake. This approach using pooled samples was previously indicated for molecular biology assays as it brings advantages by increasing population-level coverage when prevalence is low [19]. DNA was extracted from 50 mg intestinal tissue with the PureLink^®^ Genomic DNA Kit (Invitrogen, Carlsbad, CA, USA) according to manufacturer’s spin-column protocol for extraction and purification of genomic DNA from animal tissues. DNA methylation on cytosine was quantified using 5-mC DNA ELISA Kit (Zymo Research, Irvine, CA, USA) in accordance with the manufacturer’s instructions.

#### 2.7.2. Microbiome Sequencing

DNA isolation from intestinal samples was performed using the PureLink Microbiome DNA Purification Kit (Invitrogen, Waltham, MA, USA) following the manufacturer’s instructions. The PCR amplification of hypervariable regions of the 16S rDNA gene from bacteria was performed using an Ion 16S™ Metagenomics Kit (Life technologies, Carlsbad, CA, USA). PCR products derived from amplification of specific 16S rRNA gene hypervariable regions were purified using Agencourt AMPure XP DNA purification beads (Beckman Coulter, Brea, CA, USA). Concentrations of the amplified sequence libraries were calculated using Real Time PCR and a Library Quantitation kit (Life Technologies). Libraries were diluted to a 10 pM concentration prior to clonal amplification. Emulsion PCR was done using the Ion PGM™ Hi-Q™ View OT2 Kit (Life Technologies) following the supplier’s instructions. Sequencing of the amplicon libraries was performed on ION 318 chip using the Ion Torrent PGM platform and the Ion PGM™ Hi-Q™ View Sequencing Kit (Life Technologies) according to the manufacturer’s instructions.

After sequencing, the individual sequence reads were filtered by the PGM software Ion reporter in order to remove low quality and polyclonal sequences. Next, quality-approved, trimmed and filtered data were exported as sff files and processed using QIIME2. All reads were classified to the lowest possible taxonomic rank using the QIIME pipeline and the Curated Greengenes v13.5; Curated MicroSEQ (R) 16S Reference Library v2013.1 data set. 16S rRNA Operational Taxonomic Units (OTUs) were defined at ≥97% sequence homology. Alpha diversity was measured using the Shannon index metrics whereas beta diversity was calculated using Bray Curtis analysis (on a species level).

### 2.8. Statistical Analysis

All analyzes were performed in three technical replicates for each determination. Pooling samples were performed for the determination of metal, pesticides, antibiotics and DNA methylation level as the mass of intestine fish is low and to reduce the costs. The procedures were performed in agreement with the guidelines of pooling samples, using the controls and standards in accordance with each method [19]. Comparisons between groups of fish from different lakes were statistically evaluated using one-way ANOVA followed by Tukey’s multiple comparisons test and post-hoc Bonferroni test within GraphPad Prism software (Version 9.0.0, GraphPad, San Diego, CA, USA). A p value lower than 0.05 was considered statistically significant.

## 3. Results and Discussion

### 3.1. Analysis of Metals, Pesticides and Antibiotics Presence in the Gut Tissue of Fish

The presence of metals, pesticides and antibiotics were analyzed in the gut of *Carassius gibelio* from Bucharest lakes and presented in Table 2, Table 3 and Table 4, respectively. The amount of Cd found in guts was the same regardless of the lakes’ locations. It was interesting to observe that concentrations of Cr, Cu, Fe, Ni and Pb in the fish gut were at least two folds higher in Chitila and Floreasca lakes compared to Tei and Vacaresti lakes (Table 2). Cr and Cu amounts in fish guts from the Romanian lakes seemed to be similar with those found in fish collected from Lithuania’s fresh water [20], but higher than the levels in *Cyprinus carpio* from Taihu Lake of China [21].

Regarding the Fe, Ni, Pb concentrations in gut tissue from fish in the Romanian lakes, the results were significantly higher (up to 4–5 folds) than those from China and Lithuania [20,21]. The concentrations of Ca and K were higher in fish from Vacaresti and Chitila lakes compared to Tei and Floreasca samples. Zn, Mg and Na were found in the highest concentration in gut from fish collected from Chitila Lake, but Mn was highly found in Floreasca Lake (Table 2). Overall, Vacaresti Lake seemed to be an isolated environment from the other lakes, but based on metals content in fish guts seemed to the closest to Tei Lake, rather than Chitila and Floreasca lakes. The overall metal concentrations found in fish guts were not susceptible to induce major toxic effects in *Cyprinus carpio* [22].

Table 3 shows the presence of Dieldrin, 2,4′ DDE, 4,4′ DDE and 2,4′-Methoxychlor in all collected fish samples. The gut samples collected from fish in the Chitila Lake contained high levels of Aldrin, Dieldrin, 2,4′ DDE and 2,4′-Methoxychlor. It is important to mention that Aldrin and Dieldrin were banned decades ago and still persist in the environment [23] being poorly soluble and thus having a high half-life in soil. They are also highly toxic to humans and is considered as probable human carcinogens being able to accumulate in fish even if the concentration in water is below the limit of detection (<0.2 ng/g) [24]. Also, high levels of 2,4′-Methoxychlor were found in the gut samples collected from fish in the Floreasca, Vacaresti and Tei lakes. The detected pesticide levels of Aldrin, Dieldrin and 2,4′-Methoxychlor from fish in the Chitila Lake were much higher than MRL allowable to be present in food (0.01 µg/g) [25,26]. Pentachlorobenzene, Hexachlorobenzene, Pentachloroanisol, Pentachlorotioanisole, Alpha-BHC, Beta-BHC, Isodrin, Trans-chlordane, Cis-chlordane, Trans-nonachlor, Cis-nonachlor, Endosulfan sulfate, Endrin ketone and Mirex were not detected, generally (<LOD) or have values less than MRL in all fish samples. Low levels of OC and OP pesticide residues were detected in fish samples collected from Vacaresti Lake. Also, the concentration of lindane and derivatives are below the limit of quantification.

The results showed that pesticide concentrations varied with the fish location, the highest cumulative level of pesticides being in fish samples collected from Chitila and Tei lakes. Based on these results and corroborating them with different studies or regulations, we can conclude that primary predators, such as birds, can be affected by the higher content from the entire fish, while humans are exposed to lower amount of pesticides because their food is more variated and the more toxic parts are removed (gut, liver and gills) [23,27].

Buah-Kwofie and Humphries [28] reported the accumulation of organochlorine pesticides in fish and sediments even if these pesticides have been banned or severely limited worldwide. Moreover, the selective bioaccumulation tendency of the pharmaceuticals in organs is characteristic and additionally influence the toxicity and lead to specific questions and uncertainties. For instance, organochlorine pesticides were determined in the most abundant two species of fish from St. Lucia Lake and ranged between 860–5000 ng g^−1^ live weight (lw) and 390–3200 ng g^−1^ lw for *Oreochromis mossambicus* and *Clarias gariepinus*, respectively. Based on these results, they found that there are individual pesticides which have concentrations higher that the allowed maximum residue limits (MRL), while based on the estimated daily intake of these pesticides, there are concerns related with cancer or non-cancer health risks [28]. From environmental point of view, it is important to mention that the most of the organochlorine pesticides (OCPs) detected are persistent and they are of historical use.

According to European Union Council Regulation 2377/90/EC, the safe MRL values for antibiotics and other veterinary substances that can be used as veterinary drugs in animal products were established. Other regulatory agencies around the world along with Codex Alimentarius Commission have set different tolerance or MRL of these antibiotics. According to EU regulation, the MRL for Tetracycline is 0.1 µg/g, for colistin is 0.15 µg/g and for erythromycin is set to 0.2 µg/g [29]. Imipenem and vancomycin antibiotics were not detected (<LOD) in the evaluated fish samples. Also, Meropenem, Colistin sulphate, Clindamycin and Erythromycin have values less than maximum residue limits (MRL) in all fish samples.

Looking on the distribution of these antibiotics, it can be observed that the level of these antibiotics is the lowest in the fish samples from Floreasca Lake (Table 4). Comparing the samples from the four lakes, we can conclude that the gut contains similar level of tetracycline, clindamycin, and erythromycin in fish samples from Tei, Vacaresti and Chitila lakes. Moreover, the gut samples of fish collected from Tei, Vacaresti and Chitila lakes contained higher levels of tetracycline, exceeding the MRL [30]. In the current study, the LOQ of the examined antibiotics was between 0.004 μg/g and 0.06 μg/g, which was much lower than the maximum MRL level determined for some antibiotics.

The presence of antibiotics in the environment is concerning because it can assist the development of the antibiotic resistance gene (ARGs). Moreover, it is important to mention that the development of the ARGs is also enhanced by the presence of other pollutants: ions, including heavy metals, pesticides but also micro and nanoparticulate plastic materials [31,32,33,34]. Li et al. [32], for instance, evaluated the influence of the co-existence of Ag^+^ and tetracycline at environmentally relevant concentration and reveal that the respiratory activity was altered by 42%, the membrane structure was destroyed by 218%; while the relative abundances of target antibiotic resistance genes were increased with up to 92–98%. Also, it is important to note that these values highlight a potentiation of the development of the ARGs by 1.1–4.3 times comparing to the sum of the individual components. The mechanisms of action are still unclear for the potentiating effect observed for these common environmentally occurring pollutants [31,32].

### 3.2. Evaluation of Antioxidative Status

The evaluation of the most important antioxidant enzyme activities was shown in Table 5. Regarding the activity of CAT, SOD, GR and GST, the values recorded for fish from Vacaresti Lake were the highest within the all samples analyzed. The specific activity of total SOD was highest in the fish gut of individuals from Vacaresti Lake compared to that registered in fish from the other three lakes. There are two types of SOD in fish cells: the cytosolic Cu, Zn-SOD and the mitochondrial one, Mn-SOD. The highest tissue level of Mn in the fish of Vacaresti Lake could explain the highest total SOD activity. On the other hand, fish from Floreasca Lake presented the lowest total SOD activity due to the highest iron content in gut, which is in accordance with previous report [35]. The hydrogen peroxide generated in the reaction catalyzed by SOD was decomposed in the presence of CAT. As it can be seen in the Table 5, CAT activity in the fish from Chitila, Floreasca and Tei lakes was lower compared to that observed in those from Vacaresti Lake.

The most significant reduction of enzyme activity compared to levels from Vacaresti Lake was noticed for GST activity in the gut of fish from Chitila, Floreasca and Tei lakes, the level being at 17%, 21% and 13%, respectively, of that recorded in fish from Vacaresti Lake. Having an important role in detoxification, these low levels of GST might suggest an inhibition of the this enzyme’s activity, most likely explained by the high level of several heavy metals (Cr, Cu, Fe, Ni and Fe) (Table 2) that accumulated in the gut of fish found in Chitila, Floreasca and Tei lakes compared to the fish living in the protected environment of Vacaresti Lake [36,37].

**Figure 2:**
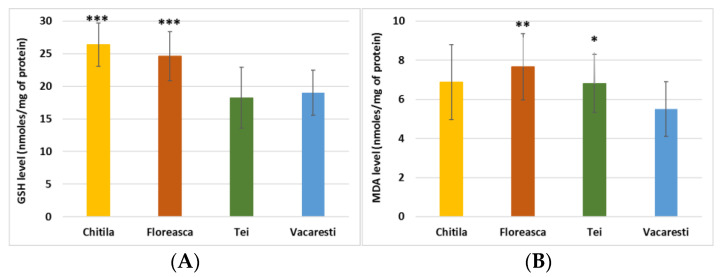
Level of (**A**) reduced glutathione (GSH) and (**B**) malondialdehyde (MDA) in the gut of *Carassus gibelio* fish from lakes in Bucharest (Chitila, Floreasca, Tei and Vacaresti). Results were expressed average values ± standard deviation (n = 7). * *p* < 0.05, ** *p* < 0.01 and *** *p* < 0.001 compared to Vacaresti Lake.

An increase of GPx activity was recorded for samples from Floreasca Lake compared to those from Vacaresti Lake. This could indicate the stimulation of this enzyme’s activity in order to remove reactive oxygen species generated by diverse aquatic pollutants. Furthermore, this could be correlated with the high level of MDA in the intestine of individuals from Floreasca Lake compared to those from Vacaresti Lake (Figure 2B).

Regarding the GR activity, the highest level was noticed also in the fish of Vacaresti Lake, compared to the level found in individuals from the other three lakes. This suggest that the transformation of oxidized glutathione in GSH occurs at the highest level in the intestine of these individuals compared to the fish from the other lakes.

GSH, the most important non-enzymatic antioxidant, showed an increased level in the samples from Chitila and Floreasca lakes compared to those from Vacaresti Lake (Figure 2A). Taking into account that GR activity was lower in the samples from these lakes, probably de novo GSH synthesis occurred, possibly in order to compensate the inhibition of GST and GPx activity. For Tei Lake, the GSH concentration the gut of fish was slightly decreased compared to that of Vacaresti Lake, suggesting that mechanisms to stimulate GSH synthesis were not activated.

The increased degree of lipid peroxidation in the gut, quantified by the level of MDA, was indicated by values higher in the gut samples of fish from the Chitila, Floreasca and Tei lakes compared to the concentration identified in the fish from Vacaresti Lake. The lowest level of MDA in the gut of fish from Vacaresti Lake could be correlated with the highest activities of GPX and GST, enzymes involved in lipid peroxides’ detoxification.

### 3.3. Evaluation of 5-mC Level in DNA of Fish Gut

**Figure 3:**
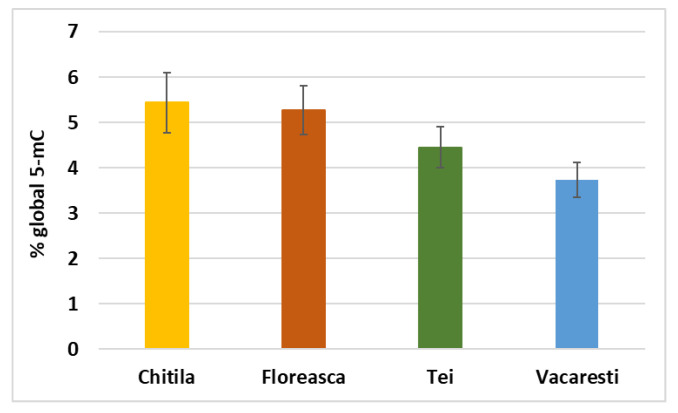
Level of 5-mC in DNA extracted from gut of fish collected from lakes in Bucharest (Chitila, Floreasca, Tei and Vacaresti). Results are expressed as average values ± standard deviation (7 fish per lake and 3 technical replicates).

The methylation of DNA represented by the covalent addition of a methyl group at carbon five of the cytosine residue (mC) can be triggered by induced in animal cells by exposure to various heavy metals (such as Cd) [38] or pesticides (such as parathion and dioxin) [39,40]. DNA hypermethylation can have a serious impact on animal and human health, as it can be associated with cancer and many other non-malignant diseases [41]. By performing an ELISA type assay, we observed the highest level of global 5-mC in the gut of fish collected from Chitila lake, and the lowest degree was recorded for Vacaresti Lake (Figure 3). However, extrapolating on a calibration curve with negative and positive controls offered by the kit, the level measured was lower that 6% for all samples tested, and it can be considered with no toxic influence on DNA structure.

### 3.4. Analysis of Gut Microbiome in Fish

Gut microbiomes of fish are complex, dynamic communities influenced by a wide array of environmental, genetic and physiological factors. The diversity of gut bacteria with regard to microbial numbers and their abundance evenness is essential for host health. Hence, lower alpha diversity (also known as intra-individual diversity) is an indicator of an unbalanced microbiome.

**Figure 4:**
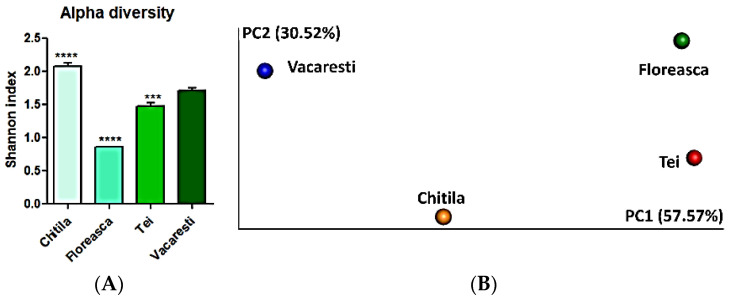
Microbiome analysis of intestinal fish samples from lakes in Bucharest (Chitila, Floreasca, Tei and Vacaresti): (**A**) alpha diversity within each sample, (**B**) beta diversity based principal component (PC) analysis (Bray-Curtis, species level). Results are presented as average values ± standard deviations (n = 7). *** *p* < 0.001 and **** *p* < 0.0001 compared to Vacaresti Lake.

Based on the diversity resistance hypothesis, a very diverse microbial community shows a higher probability to have species with an antagonistic trait against pathogens [42]. Among the samples harvested from different lakes, we showed here that fish gut samples from the Floreasca Lake exhibited the lowest alpha diversity, as measured by Shannon index calculation (Figure 4A). Conversely, samples from Chitila and Vacaresti lakes had significantly higher alpha diversity. Next, calculation of beta diversity (that shows diversity between samples) revealed that the ecological niches taken into analysis harbored distinct microbiomes, as shown in Figure 4B. Principal component (PC) 1 accounted for 57.57% of the total variation, whereas PC2 accounted for 30.52% of the variance in the bacterial communities (Figure 4B).

**Figure 5:**
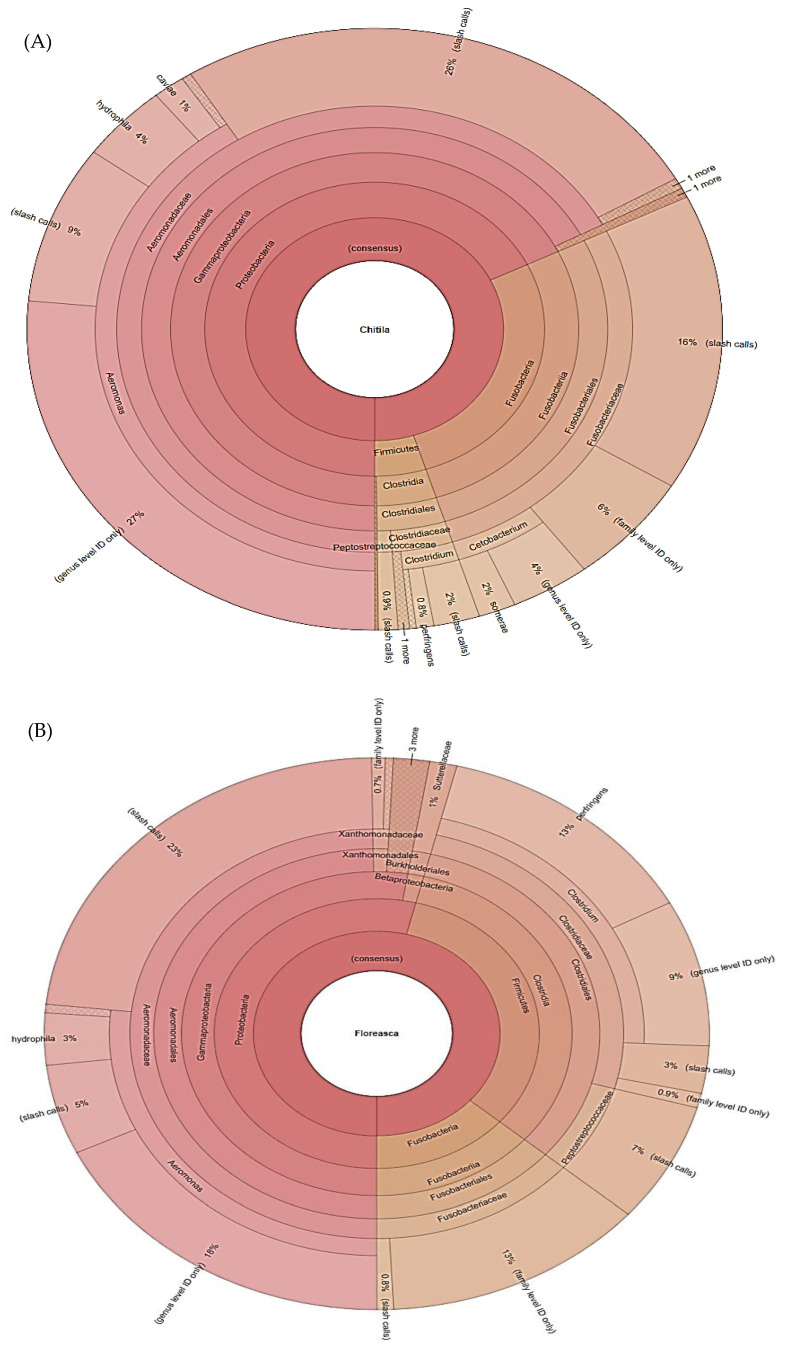
Microbiome composition in fish from Chitila (**A**) and Floreasca (**B**) lakes. OTUs represented as percentage of sequence reads. Krona plots showing 16S rRNA Operational Taxonomic Units (OTUs) abundance. The 16S rRNA amplicon sequencing was performed using Ion Torrent PGM. Results are presented as average values ± standard deviations (n = 7).

The intestinal fish samples from Chitila Lake were mainly populated by Aeromonas species (67% of reads) and members of the Fusobacteriaceae (28% of reads) family (Figure 5A). Similarly, samples from the Floreasca Lake were high in Aeromonadaceae (49% of sequencing reads), Clostridiaceae (29.5% of sequencing reads) and Fusobacteriaceae (13.8%) (Figure 5B).

**Figure 6:**
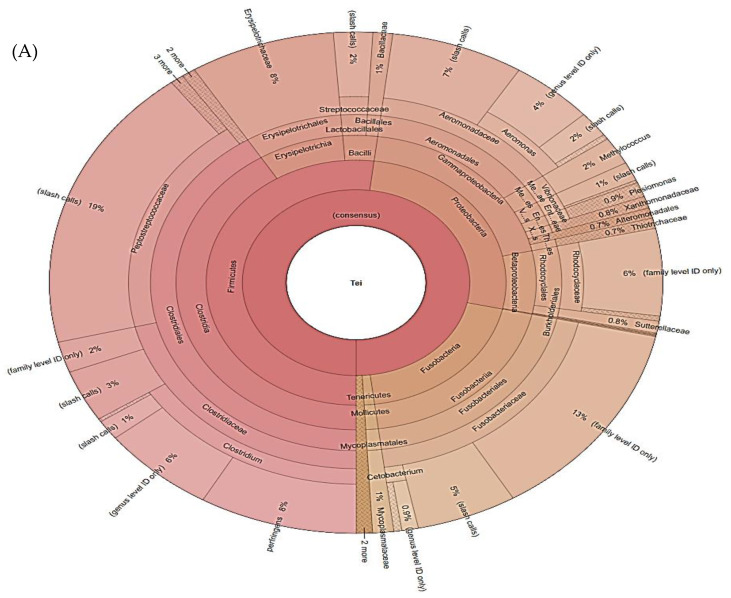

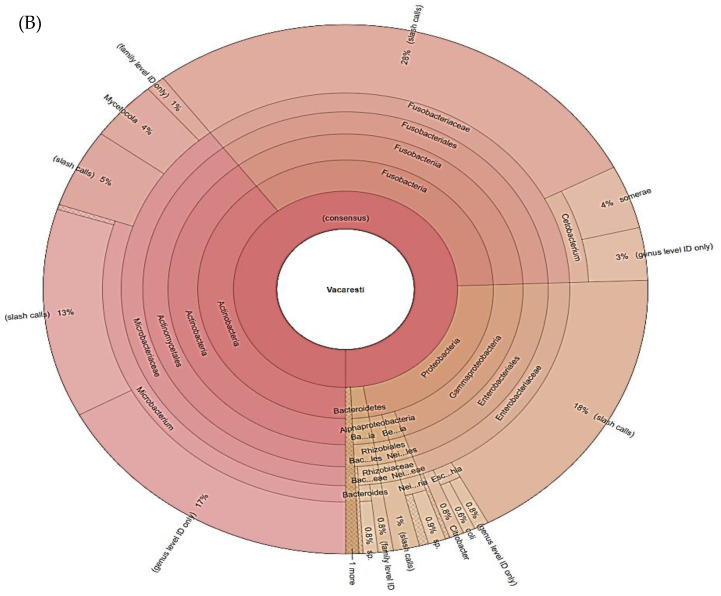
Microbiome composition in fish from Tei (**A**) and Vacaresti (**B**) lakes. OTUs represented as percentage of sequence reads. Krona plots showing 16S rRNA Operational Taxonomic Units (OTUs) abundance. The 16S rRNA amplicon sequencing was performed using Ion Torrent PGM. Results are presented as average values ± standard deviations (n = 7).

Gut samples of fish from the Tei Lake harbored a distinct microbiome pattern characterized by high abundance of Clostridiales (39% of sequencing reads) (Figure 6A). Importantly, 9% of the microbiome reads in the case of fish from Tei Lake were represented by *Clostridium perfringens*, an anaerobic gram-positive spore-forming bacillus that is associated with acute gastrointestinal infections in humans.

Other microorganisms identified in the gut of fish from Tei Lake included Fusobacteriaceae (18% of reads), Aeromonadaceae (13% of reads) and Erysipelotrichaceae (8% of reads). Importantly, Erysipelotrichaceae are members of the human gut microbiota and their enrichment has been associated with cancer, obesity and metabolic diseases [43].

Intestinal samples from the Vacaresti Lake were dominated by members of the Actinobacteria and Fusobacteria phyla. Microbacterium species were particularly dominant in samples harvested from this lake, representing 30% of the total of sequencing reads. Moreover, Fusobacteriaceae (28% of reads) and Enterobacteriaceae (18% of reads) were highly abundant in intestines of fish from the Vacaresti Lake (Figure 6B). Notably, Enterobacteriaceae are also an important member of the human gut microbiota, often associated with ailments such as inflammatory bowel disease and type 2 diabetes [44,45].

## 4. Conclusions

In the last years, anthropogenic pollution of aquatic ecosystems has increased the need for studies to identify the impact of different categories of pollutants on the species that live there. Fish are often exposed to heavily polluted water, which causes biochemical changes in the cells or changes in the whole body. Taking into consideration our results, we showed that fish from Bucharest lakes designed for leisure (Chitila, Floreasca and Tei lakes) have an increased level of oxidative stress in intestine tissue, revealed by affected antioxidant enzymes activities and GSH levels, as well as the high degree of lipid peroxidation, compared to the fish from protected environment (Vacaresti Lake). From the results obtained in this study, it was also observed that the degree of DNA methylation was slightly higher in the case of fish collected from Floreasca and Chitila lakes compared to the group of Vacaresti Lake. These changes could have serious negative consequences for the health and even survival of these organisms. In addition, these findings could be very well correlated with the presence of some heavy metals (Fe, Ni and Pb) and pesticides (Aldrin and Dieldrin) in high amount in the gut of fish with modified antioxidative status. In conclusion, our study could improve the knowledge regarding the current state of urban aquatic pollution in order to impose several environmental health measures.

## Figures and Tables

**Table 1:** Geographical coordinates of each lake selected for this study and the characteristics of *Carassius gibelio* individuals in terms of body weight and length. Results are expressed as an average value ± standard deviation of 7 fish for each lake.

Lake	Geographical Coordinates ^1^ (Latitude/Longitude)	Body Weight (g)	Body Length (cm)
Chitila	44.499861/25.998267	65.14 ± 18.38	14.93 ± 1.64
Floreasca	44.473304/26.105442	82.99 ± 11.66	16.29 ± 1.04
Tei	44.465912/26.123475	77.05 ± 9.74	16.13 ± 0.97
Vacaresti	44.399918/26.133721	86.73 ± 7.56	14.50 ± 5.50

^1^ The numbers were established using Google maps localization on the cellphone at the moment of fish collection.

**Table 2:** Level of metals in the gut of *Carassius gibelio* fish collected from lakes in Bucharest (Chitila, Floreasca, Tei and Vacaresti). Results are expressed as average values ± standard deviation (7 fish per lake and 3 technical replicates).

Metal (mg/kg of Dry Tissue)	Chitila Lake	Floreasca Lake	Tei Lake	Vacaresti Lake
Cd	0.08 ± 0.007 ^a^	0.08 ± 0.007 ^a^	<0.08 ^b^	<0.08 ^b^
Cr	1.16 ± 0.151	1.42 ± 0.184	0.20 ± 0.260	0.20 ± 0.026
Cu	6.65 ± 0.778	6.84 ± 0.800	1.7 ± 0.198	3.12 ± 0.366
Fe	414 ± 52.2	974 ± 122.8	151.4 ± 19.08	72.4 ± 9.12
Mn	37.1 ± 2.82	75.6 ± 5.74	16.18 ± 1.23	84.9 ± 4.30
Ni	3.43 ± 0.401	1.58 ± 0.184	0.76 ± 0.088	0.3 ± 0.036
Pb	2.30 ± 0.216	2.78 ± 0.262	<0.33 ^b^	<0.33 ^b^
Zn	1052 ± 105	166.6 ± 16.66	556 ± 55.6	642 ± 6.4
Ca	3836 ± 445	7704 ± 894	1220 ± 140.16	4600 ± 534
Mg	1144 ± 166	408 ± 59.2	322 ± 46.6	480 ± 69.6
Na	6283 ± 660	2892 ± 304	2372 ± 250	1832 ± 192.4
K	5928 ± 889	2602 ± 390	3592 ± 538	5806 ± 870

^a^ average value ± uncertainty value. ^b^ values lower than quantification limit (LOQ).

**Table 3:** Level of pesticides in the gut of *Carassius gibelio* fish collected from lakes in Bucharest (Chitila, Floreasca, Tei and Vacaresti). Results are expressed as average values ± standard deviation (7 fish per lake and 3 technical replicates). ND means not detected.

Pesticide (µg/g of Dry Tissue)	Chitila Lake	Floreasca Lake	Tei Lake	Vacaresti Lake
Chloroneb	0.020 ± 0.0015	0.018 ± 0.0084	0.054 ± 0.0029	0.016 ± 0.0181
Pentachlorobenzene	ND	ND	ND	ND
Alpha-BHC	0.0025 ± 0.0004	ND	0.0047 ± 0.0015	0.0013 ± 0.0025
Hexachlorobenzene	ND	ND	ND	ND
Pentachloroanisol	ND	ND	ND	ND
Beta-BHC	0.0025 ± 0.0003	ND	0.0022 ± 0.0006	0.0021 ± 0.0019
Gamma-BHC (Lindane)	0.0027 ± 0.0003	ND	ND	ND
Diazinone	0.017 ± 0.0025	0.169 ± 0.0331	0.194 ± 0.0277	0.121 ± 0.0344
Delta-BHC	0.0042 ± 0.0116	0.0014 ± 0.0014	0.0033 ± 0.0009	0.0081 ± 0.0016
Endosulfan ether	0.029 ± 0.0038	0.0031 ± 0.0009	0.021 ± 0.0087	0.0029 ± 0.0019
Chloropyriphos-methyl	0.019 ± 0.0061	0.0082 ± 0.0006	0.012 ± 0.0044	0.012 ± 0.0159
Heptachlor	0.020 ± 0.0045	0.068 ± 0.0041	0.315 ± 0.0320	0.039 ± 0.0092
Pentachlorothioanisole	ND	ND	0.0055 ± 0.0014	0.0010 ± 0.0026
Aldrin	3.429 ± 0.0188	0.018 ± 0.0031	0.058 ± 0.0169	0.012 ± 0.0138
Chlorpyrifos	0.049 ± 0.0031	0.0056 ± 0.0013	0.011 ± 0.0060	0.0078 ± 0.0032
4,4′-Dichlorobenzophenone	0.019 ± 0.0036	0.0078 ± 0.0012	0.013 ± 0.0075	0.0195 ± 0.0029
Isodrin	0.0076 ± 0.0006	0.0014 ± 0.0003	0.0035 ± 0.0015	0.0072 ± 0.0023
Heptachlor epoxide	0.026 ± 0.0047	ND	0.0097 ± 0.0020	ND
Trans-Chlordane	ND	ND	ND	ND
2,4′-DDE	0.019 ± 0.0019	0.567 ± 0.0520	1.465 ± 0.0565	0.635 ± 0.0503
Cis-Chlordane	ND	ND	ND	ND
Trans-Nonachlor	0.005 ± 0.0011	0.0021 ± 0.0004	0.0047 ± 0.0012	0.006 ± 0.0023
Chlorfenson (Ovex)	0.402 ± 0.0050	0.004 ± 0.0003	ND	ND
Dieldrin	20.456 ± 0.0475	0.090 ± 0.0104	0.293 ± 0.0404	0.056 ± 0.0244
4,4′-DDE	0.016 ± 0.0024	0.010 ± 0.0064	0.730 ± 0.0485	0.096 ± 0.0137
2,4′-DDD	0.017 ± 0.0046	0.019 ± 0.0027	0.101 ± 0.0111	0.018 ± 0.0061
Cis-Nonachlor	0.0011 ± 0.0004	ND	0.0022 ± 0.0006	ND
2,4′-DDT	0.0072 ± 0.0006	ND	0.047 ± 0.0106	0.0046 ± 0.0009
Endosulfan sulfate	ND0.0365	ND	ND	ND
Methoxychlor olefin	0.229 ± 0.0045	0.043 ± 0.0060	0.163 ± 0.0280	0.099 ± 0.0121
4,4′-DDT	0.052 ± 0.0052	0.017 ± 0.0066	0.028 ± 0.0046	0.013 ± 0.0075
2,4′-Methoxychlor	0.108 ±	2.705 ± 0.0080	6.926 ± 0.1252	1.406 ± 0.0748
Endrin ketone	ND	ND	ND	ND
Mirex	ND	ND	ND	ND
Cumulative Pesticide Level	24.9598 ± 0.0157	3.7576 ± 0.0549	10.4668 ± 0.0626	2.5835 ± 0.1371

**Table 4:** Level of antibiotics in the gut of *Carassius gibelio* fish collected from lakes in Bucharest (Chitila, Floreasca, Tei and Vacaresti). Results are expressed as average values ± standard deviation (7 fish per lake and 3 technical replicates). ND means not determined.

Antibiotic (µg/g of Dry Tissue)	Chitila Lake	Floreasca Lake	Tei Lake	Vacaresti Lake
Imipenem	ND	ND	ND	ND
Vancomycin	ND	ND	ND	ND
Meropenem	0.027 ± 0.0201	0.021 ± 0.0271	ND	0.031 ± 0.0101
Tetracycline	0.182 ± 0.0271	0.096 ± 0.0264	0.218 ± 0.0861	0.311 ± 0.0323
Colistin sulphate	0.022 ± 0.0195	ND	ND	ND
Clindamycin	0.0058 ± 0.0015	0.0031 ± 0.0019	0.0069 ± 0.0023	0.0099 ± 0.0030
Erythromycin	0.01 ± 0.0157	0.005 ± 0.0298	0.012 ± 0.0215	0.017 ± 0.0188
Cumulative drug level	0.227 ± 0.0517	0.1251 ± 0.0781	0.2369 ± 0.0976	0.3689 ± 0.0337

**Table 5:** Level of antioxidant enzymes in the gut of *Carassius gibelio* fish collected from lakes in Bucharest (Vacaresti, Floreasca, Tei and Chitila). Results are expressed as average values ± standard deviation (n = 7). Comparisons between groups of fish from different lakes (Chtila, Floreasca or Tei Lake compared to Vacaresti Lake) were statistically evaluated using one-way ANOVA followed by post-hoc Bonferroni test. *** *p* < 0.001 compared to Vacaresti Lake.

Specific Activity of Enzyme	Chitila Lake	Floreasca Lake	Tei Lake	Vacaresti Lake
Catalase (Kat/mg of total protein)	0.061 ± 0.190 ***	0.083 ± 0.025 ***	0.074 ± 0.025 ***	0.180 ± 0.025
Superoxide dismutase (U/mg of total protein)	0.077 ± 0.020 ***	0.065 ± 0.022 ***	0.091 ± 0.031 ***	0.158 ± 0.032
Glutathione reductase (U/mg of total protein)	0.017 ± 0.005 ***	0.014 ± 0.004 ***	0.024 ± 0.006 ***	0.034 ± 0.007
Glutathione peroxidase (U/mg of total protein)	93.675 ± 20.173 ***	130.161 ± 13.930 -	105.766 ± 21.316 -	119.491 ± 17.101
Glutathione S-transferase (U/mg of total protein)	0.019 ± 0.003 ***	0.025 ± 0.005 ***	0.015 ± 0.002 ***	0.116 ± 0.021

## Data Availability

Data are available upon request to the corresponding author.

## References

[1] Wu B., Wang G., Wu J., Fu Q., Liu C. (2014). Sources of heavy metals in surface sediments and an ecological risk assessment from two adjacent plateau reservoirs. PLoS ONE.

[2] Xia W., Wang R., Zhu B., Rudstam L.G., Liu Y., Xu Y., Xin W., Chen Y. (2020). Heavy metal gradients from rural to urban lakes in central China. Ecol. Process..

[3] Meftaul I.M., Venkateswarlae P., Megharaj M. (2020). Pesticides in the urban environment: A potential threat that knocks at the door. Sci. Total Environ..

[4] Carvalho I.T., Santos I. (2016). Antibiotics in the aquatic environments: A review of European scenario. Environ. Int..

[5] Lenart-Borón A., Prajsnar J., Guzil M., Borón P., Chmiel M. (2020). How much of antibiotics can enter surface water with treated wastewater and how it affects the resistance of waterborne bacteria: A case study in the Bialka river sewage treatment plant. Environ. Res..

[6] Swiacka K., Maculewicz J., Kowalska D., Caban M., Smolarz K., Swiezak J. (2022). Presence of pharmaceuticals and their metabolites in wild-living aquatic organisms—Current state of knowledge. J. Hazard. Mater..

[7] Dinu D., Marinescu D., Munteanu M.C., Staicu A.C., Costache M., Dinischiotu A. (2010). Modulatory effects of deltamethrin on antioxidant defence mechanisms and lipid peroxidation in Carassius auratus gibelio liver and intestine. Arch. Environ. Contam. Toxicol..

[8] Akinsanya B., Ayanda I.O., Fadipe A.O., Onwuka B., Saliu J.K. (2020). Heavy metals, parasitologic and oxidative stress biomarker investigations in Heterolis niloticus from Lekki Lagoon, Lagos, Nigeria. Toxicol. Rep..

[9] Wang Y., Zhao H., Liu Y., Li J., Nie X., Huang P., Xing M. (2021). Environmentally relevant concentration of sulfameth-oxazole-induced oxidative stress-cascaded damages in the intestine of grass carp and the therapeutic application of exogenous lycopene. Environ. Pollut..

[10] Nolorle-Payahua C.D., de Freitas A.S., Roesch L.F.W., Zanette Y. (2020). Environmental contamination alters the intestinal microbial community of livebearer killifish *Palloceros caudimaculatus*. Helyon.

[11] Lusková V., Lusk S., Halačka K., Vetešník L. (2010). *Carassius auratus gibelio*—The most successful invasive fish in waters of the Czech Republic. Russ. J. Biol. Invasions.

[12] Stanescu S.V. (2011). Aspects concerning the Colentina river water quality in Bucharest city (Romania). Ecoterra.

[13] Gheorghe S., Mitroi D.N., Stan M.S., Staicu C.A., Cicirma M., Lucaciu I.E., Nita-Lazar M., Dinischiotu A. (2020). Evaluation of sub-lethal toxicity of benzethonium chloride in Cyprinus carpio liver. Appl. Sci..

[14] Paoletti F., Mocali A. (1990). Determination of superoxide dismutase activity by purely chemical system based on NADP(H) oxidation. Methods Enzymol..

[15] Aebi H., Bergmeyer H.V. (1984). Catalase. Methods of Enzymatic Analysis.

[16] Habig W.H., Pabst M.J., Jakoby W.B. (1974). Glutathione S-transferases. The first enzymatic step in mercapturic acid formation. J. Biol. Chem..

[17] Beutler E. (1984). Red Cell Metabolism: A Manual of Biochemical Method.

[18] Goldberg D.M., Spooner R.J., Bergmeyer H.V. (1983). Glutathione reductase. Methods of Enzymatic Analysis.

[19] Laurin E., Thakur K., Mohr P.G., Hick P., Crane M.S.J., Gardner I.A., Moody N.J.G., Colling A., Ernst I. (2019). To pool or not to pool? Guidelines for pooling samples for use in surveillance testing of infectious diseases in aquatic animals. J. Fish. Dis..

[20] Staniskiene B., Matusevicius P., Budreckiene R., Skibniewska K.A. (2006). Distribution of heavy metals in tissues of freshwater fish in Lithuania. Pol. J. Environ. Stud..

[21] Rajeshkumar S., Li X. (2018). Bioaccumulation of heavy metals in fish species from the Meiliang Bay, Taihu Lake, China. Toxicol. Rep..

[22] Gheorghe S., Vasile G.G., Gligor C., Lucaciu I.E., Lazar M.N. (2017). Metallic elements (Cu, Zn, Ni and Mn) toxicity effects determination on a fresh water fish Cyprinus Carpio (Common Carp) laboratory acclimatized. Rev. Chim..

[23] Abrantes N., Pereira R., Goncalves F. (2010). Occurrence of pesticides in water, sediments, and fish tissues in a lake surrounded by agricultural lands: Concerning risks to humans and ecological receptors. Water Air Soil Pollut..

[24] Larson S.J., Capel P.D., Majewski M.S. (1997). Pesticides in Surface Waters: Distributions, Trends, and Governing Factors.

[25] Rahman M., Hoque M.S., Bhowmik S., Ferdousi S., Kabiraz M.P., van Brakel M.L. (2021). Monitoring of pesticide residues from fish feed, fish and vegetables in Bangladesh by GC-MS using the QuEChERS method. Heliyon.

[26] Molina-Ruiz J.M., Cieslik E., Cieslik I., Walkowska I. (2015). Determination of pesticide residues in fish tissues by modified QuEChERS method and dual-d-SPE clean-up coupled to gas chromatography-mass spectrometry. Environ. Sci. Pollut. Res..

[27] European Commission, Joint Research Centre (2003). Technical Guidance Document on Risk Assessment in support of Commission Directive 93/67/EEC on Risk Assessment for New Notified Substances, Commission Regulation (EC) No 1488/94 on Risk Assessment for existing substances. Directive 98/8/EC of the European Parliament and of the Council Concerning the Placing of Biocidal Products on the Market.

[28] Buah-Kwofie A., Humphries M.S. (2021). Organochlorine pesticide accumulation in fish and catchment sediments of Lake St Lucia: Risks for Africa’s largest estuary. Chemosphere.

[29] Canada-Canada F., de la Pena A.M., Espinosa-Mansilla A. (2009). Analysis of antibiotics in fish samples. Anal. Bioanal. Chem..

[30] Yipel M., Kurekci C., Tekeli I.O., Metli M., Sakin F. (2017). Determination of selected antibiotics in farmed fish species using LC-MS/MS. Aquac. Res..

[31] Cheng Y., Lu J.R., Fu S.S., Wang S.J., Senehi N., Yuan Q.B. (2022). Enhanced propagation of intracellular and extracellular antibiotic resistance genes in municipal wastewater by microplastics. Environ. Pollut..

[32] Li Z.H., Yuan L., Wang L., Liu Q.H., Sheng G.P. (2022). Coexistence of silver ion and tetracycline at environmentally relevant concentrations greatly enhanced antibiotic resistance gene development in activated sludge bioreactor. J. Hazard. Mater..

[33] Wu Y.Q., Wen Q.X., Chen Z.Q., Fu Q.Q., Bao H.Y. (2022). Response of antibiotic resistance to the co-exposure of sulfamethoxazole and copper during swine manure composting. Sci. Total Environ..

[34] Miao L.Z., Guo S., Wu J., Adyel T.M., Liu Z.L., Liu S.Q., Hou J. (2022). Polystyrene nanoplastics change the functional traits of biofilm communities in freshwater environment revealed by GeoChip 5.0. J. Hazard. Mater..

[35] Yadav A.K., Sinha A.K., Egnew N., Romano N., Kumar V. (2020). Potential amelioration of waterborne iron toxicity in channel catfish (Ictalurus punctatus) through dietary supplementation of vitamin C. Ecotoxicol. Environ. Saf..

[36] Leteliér M.E., Martinez M., González-Lira V., Fáundez M., Aracena-Parks P. (2006). Inhibition of cytosolic glutathione-S-transferase activity from rat liver by copper. Chem. Biol. Interact..

[37] Dobritzsch D., Grancharov K., Hermsen C., Krauss G.-J., Schaumlöffel D. (2020). Inhibitory effect of metals on animal and plant glutathione transferases. J. Trace Elem. Med. Biol..

[38] Bouwmeester M.C., Ruiter S., Lommelaars T., Sippel J., Hodemaekers H.M., van den Brandhof E.J., Pennings J.L., Kamstra J.H., Jelinek J., Issa J.P. (2016). Zebrafsh embryos as a screen for DNA methylation modifications after compound exposure. Toxicol. Appl. Pharmacol..

[39] Zhang X., Wallace A.D., Du P., Kibbe W.A., Jafari N., Xie H., Lin S., Baccarelli A., Soares M.B., Hou L. (2012). DNA methylation alterations in response to pesticide exposure in vitro. Environ. Mol. Mutagen..

[40] Fargione J.E., Tilman D. (2005). Diversity decreases invasion via both sampling and complementarity effects. Ecol. Lett..

[41] Ehrlich M. (2019). DNA hypermethylation in disease: Mechanisms and clinical relevance. Epigenetics.

[42] Lind L., Penell J., Luttropp K., Nordfors L., Syvänen A.C., Axelsson T., Salihovic S., van Bavel B., FALL T., Ingelsson E. (2013). Global DNA hypermethylation is associated with high serum levels of persistent organic pollutants in an elderly population. Environ. Int..

[43] Chen W., Liu F., Ling Z., Tong X., Xiang C. (2012). Human intestinal lumen and mucosa-associated microbiota in patients with colorectal cancer. PLoS ONE.

[44] Garrett W.S., Gallini C.A., Yatsunenko T., Michaud M., DuBois A., Delaney M.L., Punit S., Karlsson M., Bry L., Glickman J.N. (2010). Enterobacteriaceae act in concert with the gut microbiota to induce spontaneous and maternally transmitted colitis. Cell Host Microbe.

[45] Sovran B., Planchais J., Jegou S., Straube M., Lamas B., Natividad J.M., Agus A., Dupraz L., Glodt J., Da Costa G. (2018). Enterobacteriaceae are essential for the modulation of colitis severity by fungi. Microbiome.

